# Supplementary material for: The PeachSNP170K array facilitates insights into a large-scale population relatedness and genetic impacts on citrate content and flowering time
Source: Commun Biol. 2025 Jun 4;8:854. doi: 10.1038/s42003-025-08144-2 (PMC12137643; doi:10.1038/s42003-025-08144-2)
Supplement: Supplementary file 1 — Supplementary Information [file 42003_2025_8144_MOESM1_ESM.pdf]

Supplementary Figures

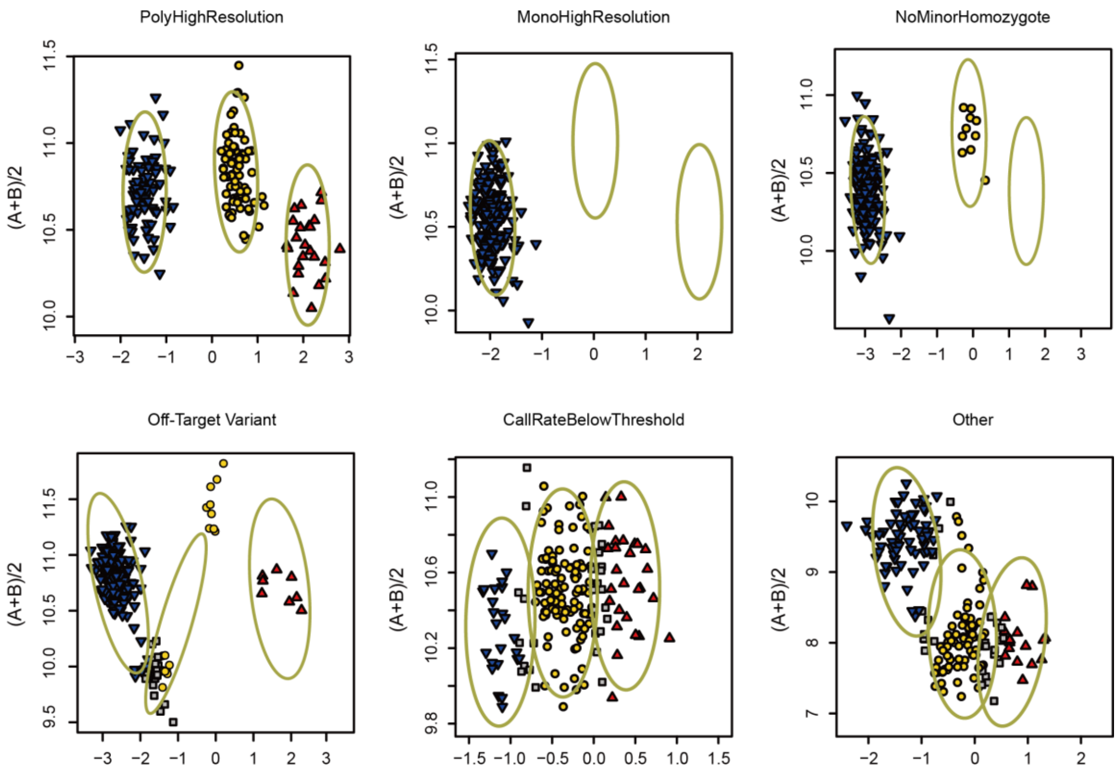

Supplementary Fig. 1 Cluster plot examples of the SNP classification categories based on the call derived from genotyping of the 192 peach accessions.

Classification categories are assigned by R package SNPolisher[77] according to the Axiom™ Genotyping Solution Data Analysis user guide, into six major types: "PolyHighResolution", "MonoHighResolution", "NoMinorHomozygote", "Off-Target Variant", "CallRateBelowThreshold," or "Other".

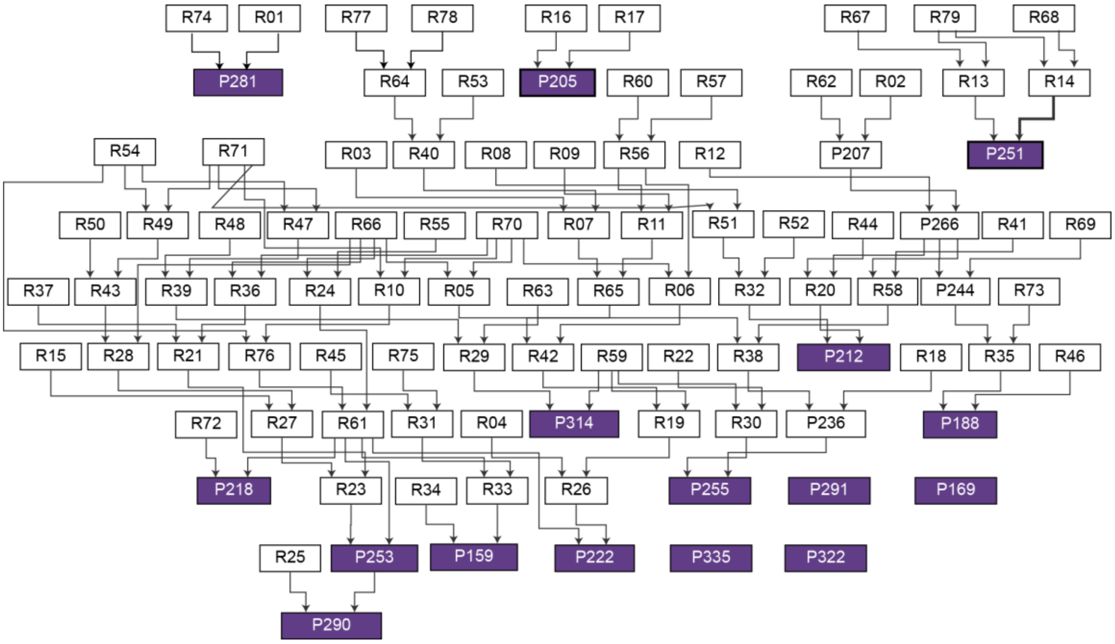

Supplementary Fig. 2 Pedigree of the peach accessions related to kinship Clr3.

The pedigree illustrates the genetic relationships among 95 peach accessions associated with kinship Clr3 (Supplementary Data 7). Rectangles denote individual peach accessions. Accessions genotyped using the PeachSNP170K array are highlighted in purple.

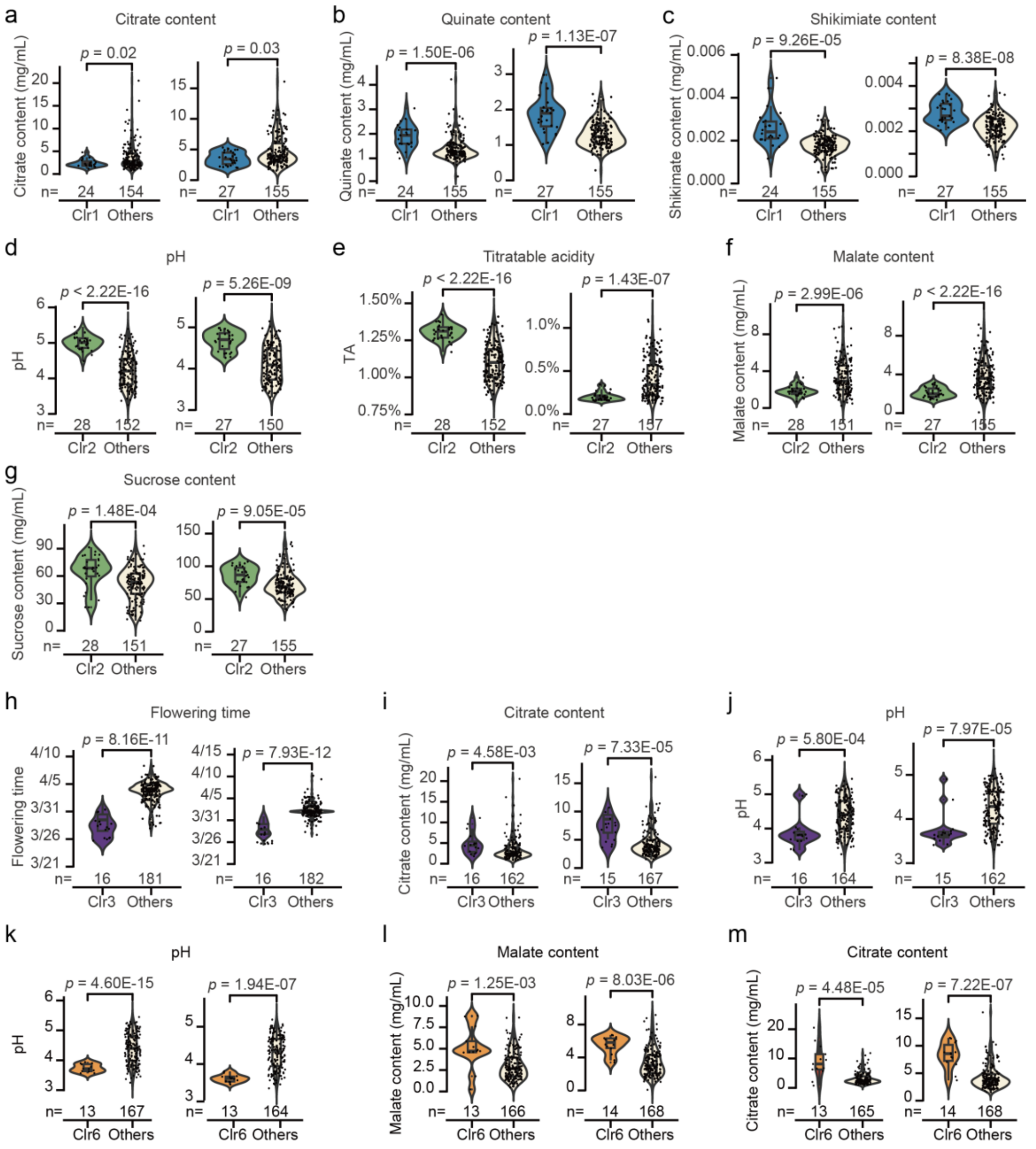

Supplementary Fig. 3 The significantly distinguished phenotypes among kinship clusters.

Comparison analysis for fruit-flavor-related traits and flowering time was carried out between Clr1 (2, 3, and 6, respectively) versus other clusters using Student's *t*-test (phenotypic distribution under normal distribution), or Mann-Whitney U test, and the significance was shown in the violin plots (a-m), respectively. Phenotypes related to fruit flavor were measured in 2016 (left panel) and 2017 (right panel). Flowering dates were recorded in 2019 (left panel) and 2020 (right panel). In the violin plots, the central line represents the median value, the bounds of the box correspond to the 25th and

75th percentiles, and the whiskers represent 1.5 x IQR (the interquartile range between the 25th and 75th percentiles).

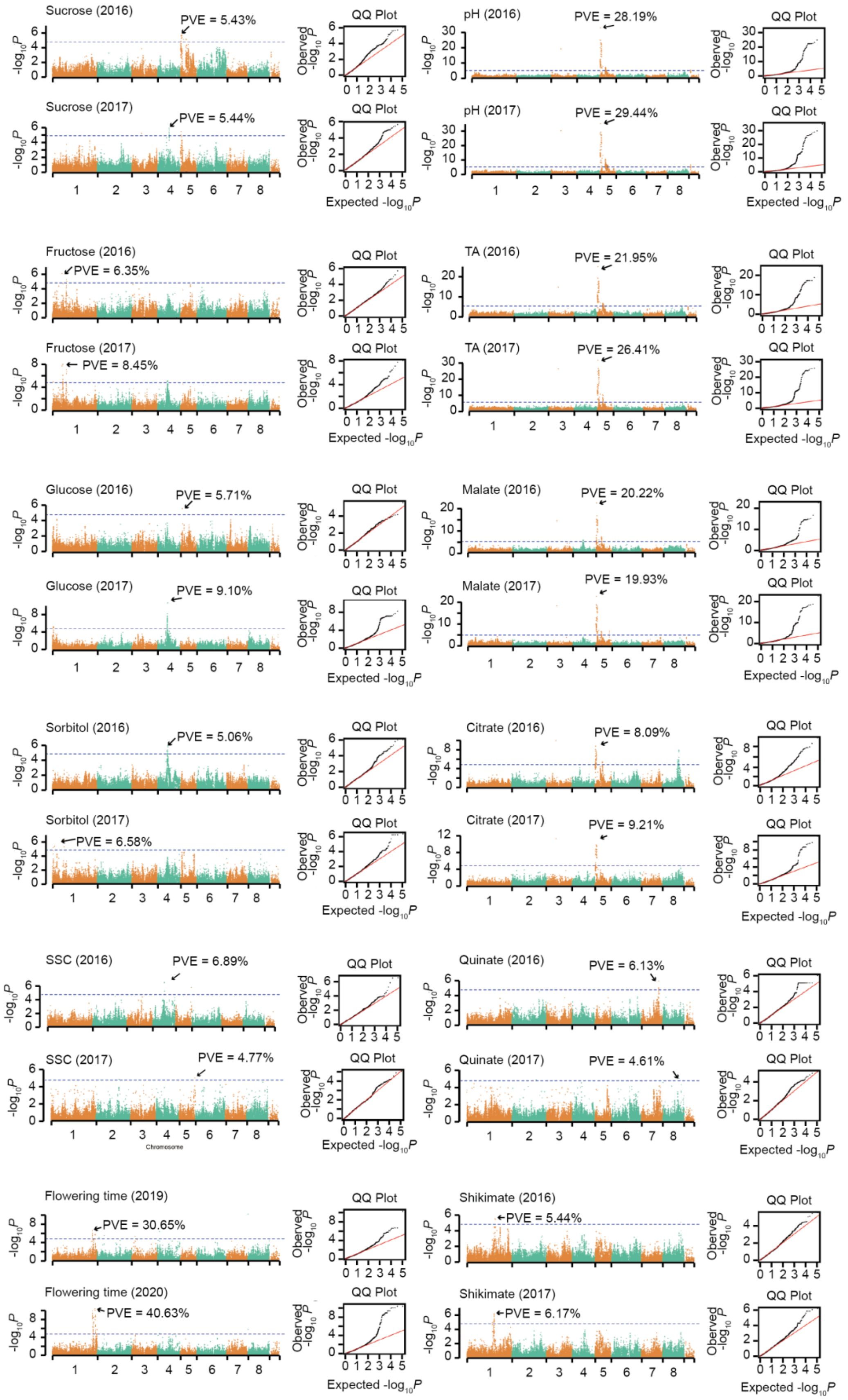
Sucrose (2016)
PVE = 5.43%
Sucrose (2017)
PVE = 5.44%
pH (2016)
PVE = 28.19%
pH (2017)
PVE = 29.44%
Fructose (2016)
PVE = 6.35%
Fructose (2017)
PVE = 8.45%
TA (2016)
PVE = 21.95%
TA (2017)
PVE = 26.41%
Glucose (2016)
PVE = 5.71%
Glucose (2017)
PVE = 9.10%
Malate (2016)
PVE = 20.22%
Malate (2017)
PVE = 19.93%
Sorbitol (2016)
PVE = 5.06%
Sorbitol (2017)
PVE = 6.58%
Citrate (2016)
PVE = 8.09%
Citrate (2017)
PVE = 9.21%
SSC (2016)
PVE = 6.89%
SSC (2017)
PVE = 4.77%
Chromosome
Quinate (2016)
PVE = 6.13%
Quinate (2017)
PVE = 4.61%
Flowering time (2019)
PVE = 30.65%
Flowering time (2020)
PVE = 40.63%
Shikimate (2016)
PVE = 5.44%
Shikimate (2017)
PVE = 6.17%
QQ Plot
-log10P
Oberved -log10P
Expected -log10P

Supplementary Fig. 4 GWAS loci associated with fruit flavor and flowering time related traits.

Titratable acid (TA), pH, and the contents of sucrose, fructose, glucose, sorbitol, malate, citrate, quinate, and shikimate were measured in 2016 and 2017, respectively. Flowering times were recorded in 2019 and 2020, respectively. The horizontal lines depict the Bonferroni-adjusted significance threshold (blue) in the Manhattan plot. The associated SNPs with the highest phenotypic variance explanation (PVE) value were shown.

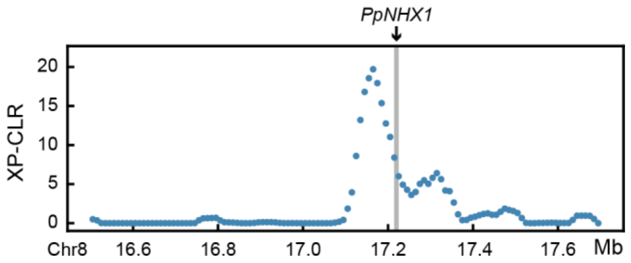

Supplementary Fig. 5 Selection signature in the comparison between Clr1 and Clr6 accessions.

The selection signature (XP-CLR) between Clr1 and Clr6 accessions is displayed in the consecutive windows overlapping with the *PpNHX1* gene.

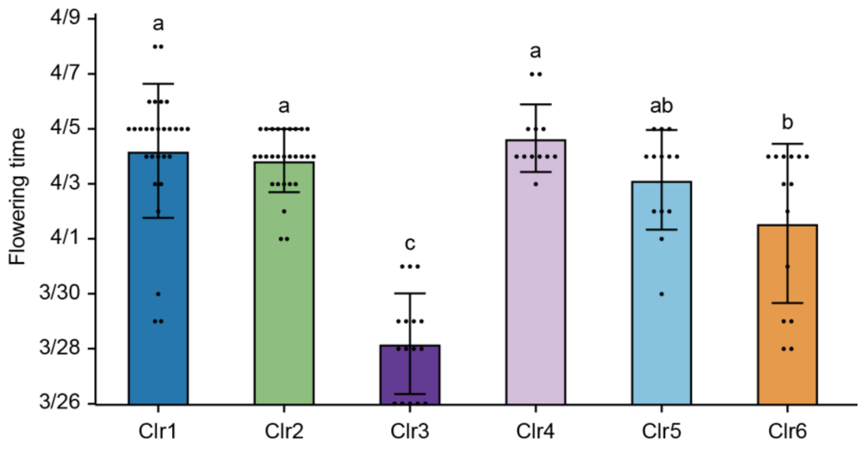

Supplementary Fig. 6   Comparison of the flowering time of the accessions from Clr1 to Clr6.

Multiple comparisons were conducted using the LSD (least significant difference) test. Data are presented as the mean ± SD. The color palette from Fig. 3d was used for the column chart representing Clr1 to Clr6, respectively.

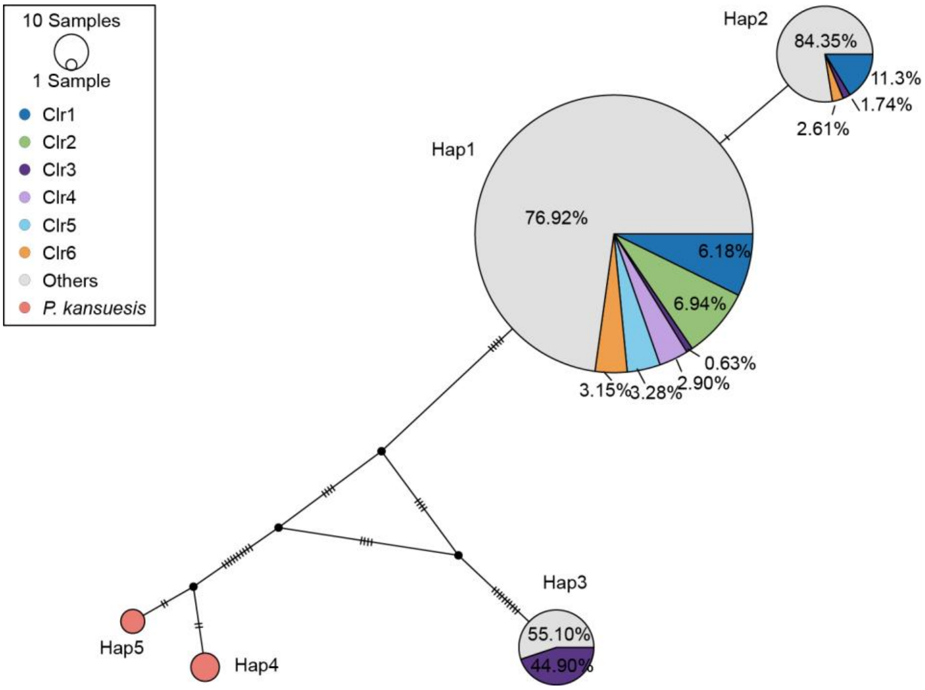

Supplementary Fig. 7 Haplotype networks of the haplotypes affecting peach flowering time.

Median-joint network analysis was used to construct the haplotype networks of the haplotypes (formed by LD block of Chr1: 45,355,352-45,435,638 bp) among the 489 accessions and with an addition of six wild peach (*P. kansuesis*) accessions, respectively. The haplotypes that were present in a single accession were excluded. The node size and the parallel lines on branches represent the relative haplotype frequency and the number of mutations, respectively. The different colored portions in each node represent the proportion of different peach accessions within each haplotype.

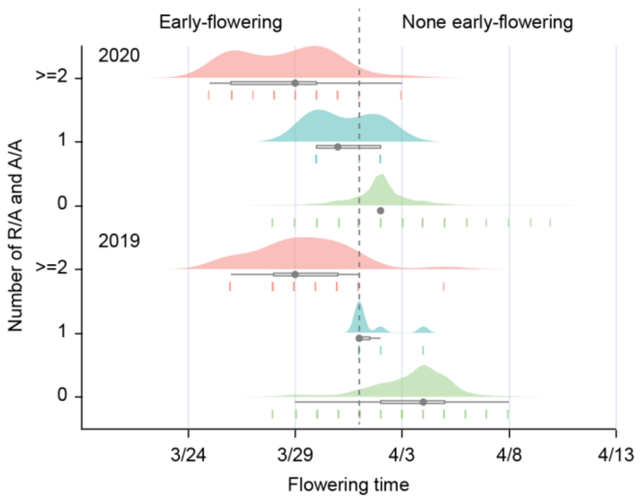

Supplementary Fig. 8 Distribution of flowering time identified by KASP markers for early-flowering traits.

Raincloud plot of early-flowering phenotype among the peach accessions with genotypes of R/A or A/A in 0, 1 and at least two SNPs. The "cloud" illustrates the data distribution, while the "rain" depicts the raw data points. The black point indicates the mean distribution value, and the black line represents the standard deviations of the distribution. The top three panels display the data collected in 2020, whereas the bottom panels show the data from 2019.

Supplementary Tables

Supplementary Table 1: Numbers of SNPs across chromosomes.

Chr	Raw SNP number	High quality SNP number	Intermediate array (620K)	PeachSNP170K array	IPSC peach 9K SNP array
Chr1	322,820	213,342	131,386	34,127	1,246
Chr2	272,064	156,412	97,050	30,143	1,551
Chr3	160,838	101,531	65,350	17,163	891
Chr4	276,710	150,111	77,714	22,100	1,781
Chr5	141,771	90,695	44,845	11,659	613
Chr6	208,649	133,479	70,977	19,861	1,034
Chr7	180,238	112,888	58,156	16,457	864
Chr8	190,829	122,117	60,667	19,155	995
Other	74,594	45,828	14,114	3,260	20
Total	1,828,513	1,126,403	620,259	173,925	8,995

Supplementary Table 2. Interval length comparison between SNPs on the PeachSNP170K array and the IPSC peach 9K SNP array.

Chr	Chromosome length (bp)	Average interval length (bp)		Maximum interval length (bp)	
		PeachSNP170K array	IPSC peach 9K SNP array	PeachSNP170K array	IPSC peach 9K SNP array
Chr1	46,877,626	1,374	37,622	61,035	527,596
Chr2	26,807,724	889	17,284	79,767	521,567
Chr3	22,025,550	1,283	24,720	48,511	393,072
Chr4	30,528,727	1,381	17,141	249,195	499,869
Chr5	18,502,877	1,587	30,184	83,141	915,779
Chr6	28,902,582	1,455	27,952	63,440	515,231
Chr7	22,790,193	1,385	26,377	93,747	455,088
Chr8	21,829,753	1,140	21,939	97,692	484,111
Other	8,986,795	2,757	449,339	-	-
Total	227,251,827	1,307	25,264	249,195	915,779

Supplementary Table 3. Annotation of SNPs on the PeachSNP170K array.

Annotation			PeachSNP170K array		Peach 9K SNP array	
			Number	Percentage	Number	Percentage
Genic	Exonic	Stop gain	445	0.26%	128	1.42%
		Stop lost	34	0.02%	11	0.12%
		Synonymous	14,308	8.23%	3,748	41.67%
		Non-synonymous	17,591	10.11%	4,757	52.88%
		Total	32,378	18.62%	8,644	96.10%
	Splicing		106	0.06%	0	0.00%
	Intronic		29,440	16.93%	241	2.68%
	Total		61,924	35.61%	8,885	98.78%
Upstream[1]			16,622	9.56%	30	0.33%
Downstream[2]			16,896	9.71%	4	0.04%
Upstream/downstream			2,830	1.63%	23	0.26%
Intergenic			75,653	43.50%	53	0.59%
Total			173,925	100%	8,995	100%

Notes:

[1] Upstream: variant located at 1 kb region upstream of a gene;

[2] Downstream: variant located at 1 kb region downstream of a gene.

Supplementary Table 4. Performance of the PeachSNP170K array.

Categorize	Number	Percentage
PolyHighResolution	132,776	76.34%
NoMinorHom	18,516	10.65%
MonoHighResolution	8,996	5.17%
Off-Target Variant	1,500	0.86%
CallRateBelowThreshold	1,993	1.15%
Other	10,144	5.83%
Total	173,925	100.00%

Supplementary Table 5. Inbreeding coefficients (F-values) in peach landraces and cultivars.

Peach Group	Mean F-value	Standard Deviation (SD)
Landrace	0.345	0.240
Cultivar (Europe)	0.075	0.229
Cultivar (America)	0.111	0.184
Cultivar (South China)	-0.068	0.180
Cultivar (North China)	0.094	0.156
Cultivar (China)	0.078	0.148
Cultivars from other Asian countries	0.098	0.307

Supplementary Table 6. The genotype, number of the accessions, and the average citrate content of each haplotype.

Position	Hap1	Hap2	Hap3	Hap4	Hap5
Chr8:17,220,030	T	T	T	T	G
Chr8:17,220,127	C	T	T	C	T
Chr8:17,220,240	C	T	C	T	C
No. of accessions (2016)	604	109	48	37	8
Percentage	74.94%	13.52%	5.96%	4.59%	0.99%
Mean content of citrate (mg mL^{-1})	2.99	5.69	3.36	3.43	1.76

Supplementary Table 7. The genotype, number of the accessions, and the average flowering date of each haplotype.

Position	Allele (Reference)	Allele (Alternate)	Hap1	Hap2	Hap3
Chr1:45,355,352	A	G	A	A	G
Chr1:45,355,677	C	G	C	C	G
Chr1:45,355,882	G	T	G	G	T
Chr1:45,356,724	A	G	A	A	G
Chr1:45,358,864	T	C	T	T	C
Chr1:45,365,756	T	C	T	T	C
Chr1:45,375,955	G	A	G	G	A
Chr1:45,379,885	T	G	T	T	G
Chr1:45,379,925	G	C	G	G	C
Chr1:45,393,095	C	G	C	C	G
Chr1:45,397,405	A	G	A	A	G
Chr1:45,398,583	T	C	T	T	C
Chr1:45,400,035	A	G	A	A	G
Chr1:45,402,011	A	T	A	A	T
Chr1:45,404,038	A	G	A	A	G
Chr1:45,408,105	C	T	C	C	T
Chr1:45,414,874	C	T	C	C	T
Chr1:45,426,756	C	T	C	T	C
Chr1:45,427,123	T	C	T	T	C
Chr1:45,435,638	G	T	G	G	T
No. of accessions (2019)			782	115	44
Percentage			83.10%	12.22%	4.68%
Mean bloom date (2019)			Apr. 3th	Apr. 3th	Mar. 29th
No. of accessions (2020)			774	115	47
Percentage			82.69%	12.29%	5.02%
Mean bloom date (2020)			Apr. 1st	Apr. 1st	Mar. 28th

Supplementary Table 8. KASP markers assisted selection for early-flowering peaches.

Location	R	A	Type	Sequence
Chr1: 45,355,677	C	G	P1	GAAGGTGACCAAGTTCATGCTGCCACCTCGAAGCACAGC
			P2	GAAGGTCGGAGTCAACGGATTGCCACCTCGAAGCACAGG
			P3	TTGTACTAAGCTTGTGGGTGAGTC
Chr1: 45,402,011	A	T	P1	GAAGGTGACCAAGTTCATGCTTTTAGACCTGCTATGATTTTCAACATA
			P2	GAAGGTCGGAGTCAACGGATTTTTAGACCTGCTATGATTTTCAACATT
			P3	ACTGTCGTTGCTCATCTTGTACTT
Chr1: 45,427,123	T	C	P1	GAAGGTGACCAAGTTCATGCTTTGACAGACTTTGACGGTAGGACC
			P2	GAAGGTCGGAGTCAACGGATTTTGACAGACTTTGACGGTAGGACT
			P3	CTGAAATGATCCCCCAACTATTGC

Note: FAM and HEX dyes were utilized for the P1 and P2 primers, respectively.
