# Supplementary material for: The PeachSNP170K array facilitates insights into a large-scale population relatedness and genetic impacts on citrate content and flowering time
Source: Commun Biol. 2025 Jun 4;8:854. doi: 10.1038/s42003-025-08144-2 (PMC12137643; doi:10.1038/s42003-025-08144-2)
Supplement: Supplementary file 2 — Description of Additional Supplementary Materials [file 42003_2025_8144_MOESM2_ESM.pdf]

Description of Additional Supplementary Files

File name: Supplementary Data 1

Description: Genomic data of 96 peach accessions downloaded from three previous studies.

File name: Supplementary Data 2

Description: 192 peach accessions used for SNP variants validation.

File name: Supplementary Data 3

Description: Genotype comparison between the PeachSNP170K array and re-sequencing data.

File name: Supplementary Data 4

Description: 489 peach accessions genotyped by the PeachSNP170K array.

File name: Supplementary Data 5

Description: Kinship coefficient between accessions (kinship coefficient greater than 0.45 was shown).

File name: Supplementary Data 6

Description: Peach accessions in 25 kinship clusters.

File name: Supplementary Data 7

Description: The peach accessions related to kinship Clr3 based on the recorded pedigree.

File name: Supplementary Data 8

Description: The IBD regions shared by peach accessions within each cluster.

File name: Supplementary Data 9

Description: SNPs significantly associated with agronomic traits.

File name: Supplementary Data 10

Description: Publicly available loci associated with agronomic traits in peach.

File name: Supplementary Data 11

Description: Source data used for all figures.

File name: Supplementary Data 12

Description: SNP-based and pedigree-based kinship coefficients between accessions (source data for Fig. 3b).
