# Supplementary material for: The PeachSNP170K array facilitates insights into a large-scale population relatedness and genetic impacts on citrate content and flowering time
Source: Commun Biol. 2025 Jun 4;8:854. doi: 10.1038/s42003-025-08144-2 (PMC12137643; doi:10.1038/s42003-025-08144-2)
Supplement: Supplementary file 15 — Reporting Summary [file 42003_2025_8144_MOESM15_ESM.pdf]

nature portfolio

Corresponding author(s): COMMSBIO-24-5716B
Last updated by author(s): Apr 13, 2025

Reporting Summary

Nature Portfolio wishes to improve the reproducibility of the work that we publish. This form provides structure for consistency and transparency in reporting. For further information on Nature Portfolio policies, see our Editorial Policies and the Editorial Policy Checklist.

Statistics

For all statistical analyses, confirm that the following items are present in the figure legend, table legend, main text, or Methods section.

n/a	Confirmed	
☐	☒	The exact sample size (*n*) for each experimental group/condition, given as a discrete number and unit of measurement
☐	☒	A statement on whether measurements were taken from distinct samples or whether the same sample was measured repeatedly
☐	☒	The statistical test(s) used AND whether they are one- or two-sided<br>*Only common tests should be described solely by name; describe more complex techniques in the Methods section.*
☐	☒	A description of all covariates tested
☐	☒	A description of any assumptions or corrections, such as tests of normality and adjustment for multiple comparisons
☐	☒	A full description of the statistical parameters including central tendency (e.g. means) or other basic estimates (e.g. regression coefficient) AND variation (e.g. standard deviation) or associated estimates of uncertainty (e.g. confidence intervals)
☐	☒	For null hypothesis testing, the test statistic (e.g. *F*, *t*, *r*) with confidence intervals, effect sizes, degrees of freedom and *P* value noted<br>*Give P values as exact values whenever suitable.*
☒	☐	For Bayesian analysis, information on the choice of priors and Markov chain Monte Carlo settings
☒	☐	For hierarchical and complex designs, identification of the appropriate level for tests and full reporting of outcomes
☐	☒	Estimates of effect sizes (e.g. Cohen's *d*, Pearson's *r*), indicating how they were calculated

Our web collection on statistics for biologists contains articles on many of the points above.

Software and code

Policy information about availability of computer code

Data collection	Solid soluble content: digital hand-held refractometer (ATAGO Pocket refractometer, PAL-1);<br>pH: pH electrode (Sartorius, PB-10);<br>Titratable acid: One ClickTM Titration (METTLER TOLEDO, T50)<br>Organic acid and sugar content: high-performance liquid chromatography (HPLC) analysis system(Shimadzu, LC-20A);<br>The qRT-PCR data: StepOnePlusTM Real-time PCR System (Thermo Fisher)
Data analysis	1. SNP identification was employed using BWA (v0.7.12).<br>2. SNP performance was assessed using Affymetrix Genotyping Console software (v.4.2);<br>3. SNP array hybridization intensity data, clustering, and genotype calling were conducted using Affymetrix Power Tools (v1.15.0) and the R package SNPolisher (v1.5.2);<br>4. Kinship coefficients were calculated using GEMMA (v0.98.1-0);<br>5. Pairwise IBD blocks were identified using Beagle (v5.1) RefinedIBD (v17Jan20.102);<br>6. GWAS analysis was applied using FaST-LMM (v2.06.20130802);<br>7. Selective sweep was analyzed using XPCLR64 (v1.1);

For manuscripts utilizing custom algorithms or software that are central to the research but not yet described in published literature, software must be made available to editors and reviewers. We strongly encourage code deposition in a community repository (e.g. GitHub). See the Nature Portfolio guidelines for submitting code & software for further information.

Data

Policy information about availability of data

All manuscripts must include a data availability statement. This statement should provide the following information, where applicable:

- Accession codes, unique identifiers, or web links for publicly available datasets
- A description of any restrictions on data availability
- For clinical datasets or third party data, please ensure that the statement adheres to our policy

Genomic data of 96 peach accessions were obtained from the Short Read Archive (https://www.ncbi.nlm.nih.gov/sra/) under accession numbers SRA053230 and SRA073649, as well as from NCBI project PRJNA310042 (https://www.ncbi.nlm.nih.gov/bioproject/?term=PRJNA310042). The cDNA sequence of PpNHX1 was available at https://www.ncbi.nlm.nih.gov/nuccore/OR713114.1/.

Research involving human participants, their data, or biological material

Policy information about studies with human participants or human data. See also policy information about sex, gender (identity/presentation), and sexual orientation and race, ethnicity and racism.

Reporting on sex and gender	This information is not applicable to this study.
Reporting on race, ethnicity, or other socially relevant groupings	This information is not applicable to this study.
Population characteristics	This information is not applicable to this study.
Recruitment	This information is not applicable to this study.
Ethics oversight	This information is not applicable to this study.

Note that full information on the approval of the study protocol must also be provided in the manuscript.

Field-specific reporting

Please select the one below that is the best fit for your research. If you are not sure, read the appropriate sections before making your selection.

☒ Life sciences ☐ Behavioural & social sciences ☐ Ecological, evolutionary & environmental sciences

For a reference copy of the document with all sections, see nature.com/documents/nr-reporting-summary-flat.pdf

Life sciences study design

All studies must disclose on these points even when the disclosure is negative.

Sample size	Sample size for each experiments is indicated in the figures or corresponding figure legends.
Data exclusions	This is not applicable.
Replication	For agronomic trait evaluation, ten fruits per plant were pooled for analysis.<br>For transient overexpression assay, the experiments were repeated at least three times, with four to five fruits taken from each test group.
Randomization	Fifty peach accessions were randomly selected to re-sequence for assess the SNP genotyping accuracy.
Blinding	This is not applicable.

Reporting for specific materials, systems and methods

We require information from authors about some types of materials, experimental systems and methods used in many studies. Here, indicate whether each material, system or method listed is relevant to your study. If you are not sure if a list item applies to your research, read the appropriate section before selecting a response.

Materials & experimental systems

n/a	Involved in the study
☒	☐ Antibodies
☒	☐ Eukaryotic cell lines
☒	☐ Palaeontology and archaeology
☒	☐ Animals and other organisms
☒	☐ Clinical data
☒	☐ Dual use research of concern
☐	☒ Plants

Methods

n/a	Involved in the study
☒	☐ ChIP-seq
☒	☐ Flow cytometry
☒	☐ MRI-based neuroimaging

Dual use research of concern

Policy information about dual use research of concern

Hazards

Could the accidental, deliberate or reckless misuse of agents or technologies generated in the work, or the application of information presented in the manuscript, pose a threat to:

No	Yes
☒	☐ Public health
☒	☐ National security
☒	☐ Crops and/or livestock
☒	☐ Ecosystems
☒	☐ Any other significant area

Experiments of concern

Does the work involve any of these experiments of concern:

No	Yes
☒	☐ Demonstrate how to render a vaccine ineffective
☒	☐ Confer resistance to therapeutically useful antibiotics or antiviral agents
☒	☐ Enhance the virulence of a pathogen or render a nonpathogen virulent
☒	☐ Increase transmissibility of a pathogen
☒	☐ Alter the host range of a pathogen
☒	☐ Enable evasion of diagnostic/detection modalities
☒	☐ Enable the weaponization of a biological agent or toxin
☒	☐ Any other potentially harmful combination of experiments and agents

Plants

Seed stocks	The peach accessions in this study were obtained from National Germplasm Repository of Peach and Strawberry (Beijing, China).
Novel plant genotypes	This is not applicable.
Authentication	This is not applicable.
